# Clinical, genotypic, and neuropsychological profile in a series of patients with Niemann-Pick type C disease

**DOI:** 10.3389/fneur.2025.1542310

**Published:** 2025-02-26

**Authors:** Rita dos Santos Mendes, Daniel Almeida do Valle, Tiago dos Santos Bara, Vanessa Furlin, Michelle da Silva Zeny, Mara Lúcia Schmitz Ferreira Santos, Mara L. Cordeiro

**Affiliations:** ^1^Faculdades Pequeno Principe, Curitiba, Brazil; ^2^Hospital Pequeno Príncipe, Curitiba, Brazil; ^3^Instituto de Pesquisa Pelé Pequeno Principe, Curitiba, Brazil; ^4^Department of Psychiatry and Biobehavioral Sciences, David Geffen School of Medicine, University of California Los Angeles (UCLA), Los Angeles, Los Angeles, CA, United States

**Keywords:** Niemann-Pick disease type C, neuropsychological assessment, mental disorders, metabolic rare disorders, genetic association studies, NPC

## Abstract

**Background:**

Niemann-Pick type C (NPC) disease is a rare neurodegenerative disorder with a wide spectrum of clinical manifestations and genetic variability. This cross-sectional study aimed to comprehensively describe the neuropsychological impact of NPC and investigate its correlation with specific genotypes.

**Results:**

Eight patients from six unrelated families were included in this study. Their age at symptom onset ranged between 2 and 16 years, with all patients presenting with ataxia, dysarthria, and cognitive impairment. Following the initiation of miglustat treatment, five patients showed a decrease in the Scale for the Assessment and Rating of Ataxia (SARA) score, whereas three demonstrated subsequent increases. Five patients underwent brain magnetic resonance imaging scans, revealing white matter abnormalities and/or brain volumetric reduction in three cases. Despite the small sample size, the overall cognitive performance of the cohort was significantly below the average. The Family Environment Scale highlighted positive structural patterns, particularly regarding Personal Growth and System Maintenance. Genetic analysis identified five mutations in the *NPC1* gene that correlated with the severity of impairments and clinical outcomes.

**Conclusion:**

This study indicated a consistent association between cognitive and behavioral impairments, with severity correlating with age and specific genetic variants. Notably, one subgroup showed a higher prevalence of psychotic and behavioral symptoms, suggesting a potential link with specific genetic variants.

## 1 Introduction

Niemann-Pick type C (NPC) is a rare hereditary disorder caused by mutations in *the NPC1* or *NPC2* genes. The clinical manifestations of NPC vary significantly, making early diagnosis challenging (1). This disease is characterized by defects in lipid metabolism and lysosomal storage, leading to cholesterol and sphingolipids accumulation in lysosomes (2). Clinical heterogeneity in patients with NPC is evident through differences in age at onset, presenting signs, and patterns of organ system involvement (3).

Genotype–phenotype relationships for *NPC1* variants are increasingly being identified, enabling greater predictability of symptom severity and disease progression (3). Additionally, broader utilization of whole-exome sequencing allows for identification of non-classical NPC phenotypes, ranging between more severe and milder forms (4).

Cognitive impairment is a primary symptom of NPC, with some studies suggesting its presence in nearly all patients with NPC (5). Nevertheless, limited data are available regarding the neuropsychological profiles of patients with NPC, particularly adults, and their correlation with specific genotypes (6, 7). Conducting further assessments of cognitive impairment and family functioning is crucial for gaining a comprehensive understanding of the challenges faced by patients. Such assessments can help tailor cognitive remediation strategies and facilitate provision of appropriate support in educational and occupational settings (7).

This study aimed to provide a comprehensive analysis of clinical, genetic, and psychological data to contribute to a deeper understanding of NPC.

## 2 Methods

This cross-sectional, observational, descriptive study was conducted at Pequeno Príncipe Children’s Hospital and approved by our Ethics Committee (Approval #CAAE 31880620.9.0000.0097). All experiments were performed in accordance with the guidelines and regulations of the Brazilian National Commission of Health (Commission of Ethics in Human Research-*CEP/CONEP*). The participants and/or their parents provided formal written consent for the use of all data for publication of this study.

We included 8 participants with a pathogenic or likely pathogenic variant in homozygosity or compound heterozygosity in *the NPC1* or *NPC2* gene. Genetic testing was conducted for the new-generation sequence of genes *NPC1* and *NPC2*. Buccal swab samples were collected, and deoxyribonucleic acid (DNA) was extracted from the samples for genetic analysis using probes for the target regions. Next-generation sequencing was performed using Illumina technology, and alignment and variant identification were performed based on bioinformatics protocols using the GRCh38 human genome as a reference. Potential pathogenic variants and regions with inadequate sequencing depth were confirmed by automated Sanger sequencing, which was conducted using a genetic analyzer. Variants were described according to the nomenclature recommended by the Human Genomic Variation Society.

Novel variants were classified according to the guidelines of the American College of Medical Genetics and Genomics (8) based on very low allele frequency, compound heterozygosity with a pathogenic variant, residue evolutionary conservation, and biochemical results. New variants were checked using the Human Gene Variant and ClinVar databases. Mutations were grouped according to their type (missense or non-missense). The pathogenicity of novel missense mutations was predicted using in silico analysis. The variants of uncertain significance were reclassified in some patients after evaluation of clinical aspects, analysis of segregation and other family members.

Regarding the clinical severity assessment of patients, the 5-domain NPC Clinical Severity Scale (NPCCSS) score (9) and Scale for the Assessment and Rating of Ataxia (SARA) were used (10).

The estimated full-scale intelligence quotient (FISQ) was assessed using the Wechsler Abbreviated Scale of Intelligence (WASI) (11). The WASI comprises four subtests, two verbal and two performance scales: Vocabulary, Similarities, Block Design, and Matrix Reasoning. The raw scores obtained using the four subtests were converted into scaled scores with an interpretation based on the standard score system: A mean of 100 and standard deviation of 15. Notably, “Average,” “Below average“, and “Borderline” and “Extremely Low” scores were considered those: in the range of 90–109, 80–89, and 70–79 and below 69, respectively. Scores in the range of 110–119 and 120–129 and above 130 were considered “High Average” and “Superior” and “Very Superior,” respectively (11).

The Adult Self-Report (ASR) and Child Behavior Check List (CBCL) are part of a set of scales used to monitor behavioral and emotional problems in individuals aged 18–80 years and 30 months-18 years, respectively; it is a reliable and robust instrument, with a sensitivity of 80% and specificity of 95% (12). The raw score is converted into T-scores using the Assessment Data Manager (ADM) software and quantified across dimensions, such as anxiety/depression, withdrawal, somatic complaints, social problems, thinking problems, attention problems, rule-breaking behavior, aggressive behavior, depressive problems, anxiety problems, somatic problems, attention-deficit/hyperactivity disorder (ADHD), oppositional defiant disorder, and conduct disorder. Additionally, the ASR and CBCL provides a total problem score and scores for internalizing and externalizing problems (13).

## 3 Results

Eight patients with NPC were identified from six unrelated families, including four (50%) female and four (50%) male individuals, aged between 5 and 36 years. Table 1 summarizes the clinical, genetic, and neuropsychological findings of all the patients.

**Table 1:** Clinical, genetic, and neuropsychological data of the patients with NPC.

		P1	P2	P3	P4	P5	P6	P7	P8
Sex		F	F	M	M	M	F	F	M
Age		36	23	22	19	23	24	5	17
Age at diagnosis (years)		27	13	12	14	12	10	4	14
Phenotype classification		Juvenile	Juvenile	Juvenile	Juvenile	Juvenile	Juvenile	Late infantile	Juvenile
Family		1	1	1	2	3	4	5	6
Gene		*NPC1*	*NPC1*	*NPC1*	*NPC1*	*NPC1*	*NPC1*	*NPC1*	*NPC1*
Variant 1	Variation	c.3019C > G	c.3019C > G	c.3019C > G	c.3104C > T	c.3104C > T	IVS24 + 2 T > G	c.3104C > T	c.2972_2973delAG
	Consequence	P1007A	P1007A	P1007A	A1035V	A1035V	−	A1035V	Q991fs
	Type	Missense	Missense	Missense	Missense	Missense	Splicing	Missense	Frameshift
Variant 2	Variation	c.3019C > G	c.3019C > G	c.3019C > G	c.3019C > G	c.3019C > G	c.3019C > G	c.3689del	c.3019C > G
	Consequence	P1007A	P1007A	P1007A	P1007A	P1007A	P1007A	L1230fs	P1007A
	Type	Missense	Missense	Missense	Missense	Missense	Missense	Frameshift	Missense
Age at first neurological symptoms		16	14	12	6	8	5	2	2
Age at onset of ataxia		16	14	12	10	12	6	2	2
Epilepsy		−	−	−	+	+	+	+	+
NPCCSS		9	8	3	7	4	18	25	25
SARA		NA	19	7	33	NA	NA	40	40
WASI		40	55	60	55	NA	46	44	NA
Internalizing problems		48	44	40	44	73	51	53	NA
Externalizing problems		59	39	45	29	93	57	37	NA
Total problems		54	40	41	40	86	58	49	NA

NA: Testing could not be performed; P8 was too compromised and hospitalized in a psychiatric clinic. M, male; F, female; NPCCSS, Niemann-Pick disease type C Clinical Severity Scale; SARA, Scale for the Assessment and Rating of Ataxia; WASI, Wechsler Abbreviated Scale of Intelligence.

The age at the onset of neurological symptoms ranged between 2 and 16 years; all patients exhibited ataxia, dysarthria, and cognitive impairment. Comorbidities included epilepsy and psychiatric disorders, with six patients using psychotropic drugs (such as antipsychotics and antidepressants) and five taking anticonvulsant drugs.

**Figure 1:**
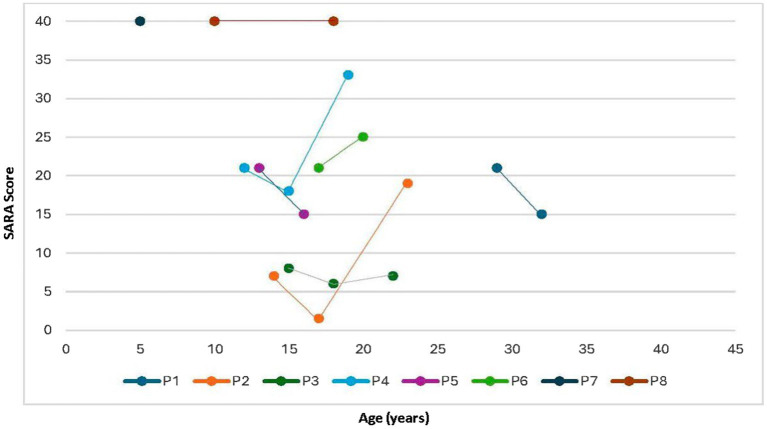
Evolution of SARA scores according to age. SARA, Scale for the Assessment and Rating of Ataxia.

SARA scores were comparatively assessed in some patients across consultations in 2013, 2016, and 2023. The follow-up has been performed since 2010. Patients P1, P2, P3, P4 and P5 have been followed up for a minimum of 7 years. Patients P6 and P7 were followed up for 3 years and 5 years, respectively. Some patients exhibited a ceiling effect during the initial assessment (P7 and P8). Following the commencement of miglustat treatment, five patients (P1, P2, P3, P4, and P5) showed a decrease in the SARA score, whereas one (P6) exhibited an increase in score despite treatment initiation. A follow-up evaluation of three of these patients (P2, P3, and P4) revealed that one patient (P3) had a slight increase in the score, whereas the other two (P2 and P4) demonstrated substantial increases, with scores surpassing their values before treatment. These findings are illustrated in Figure 1.

**Figure 2:**
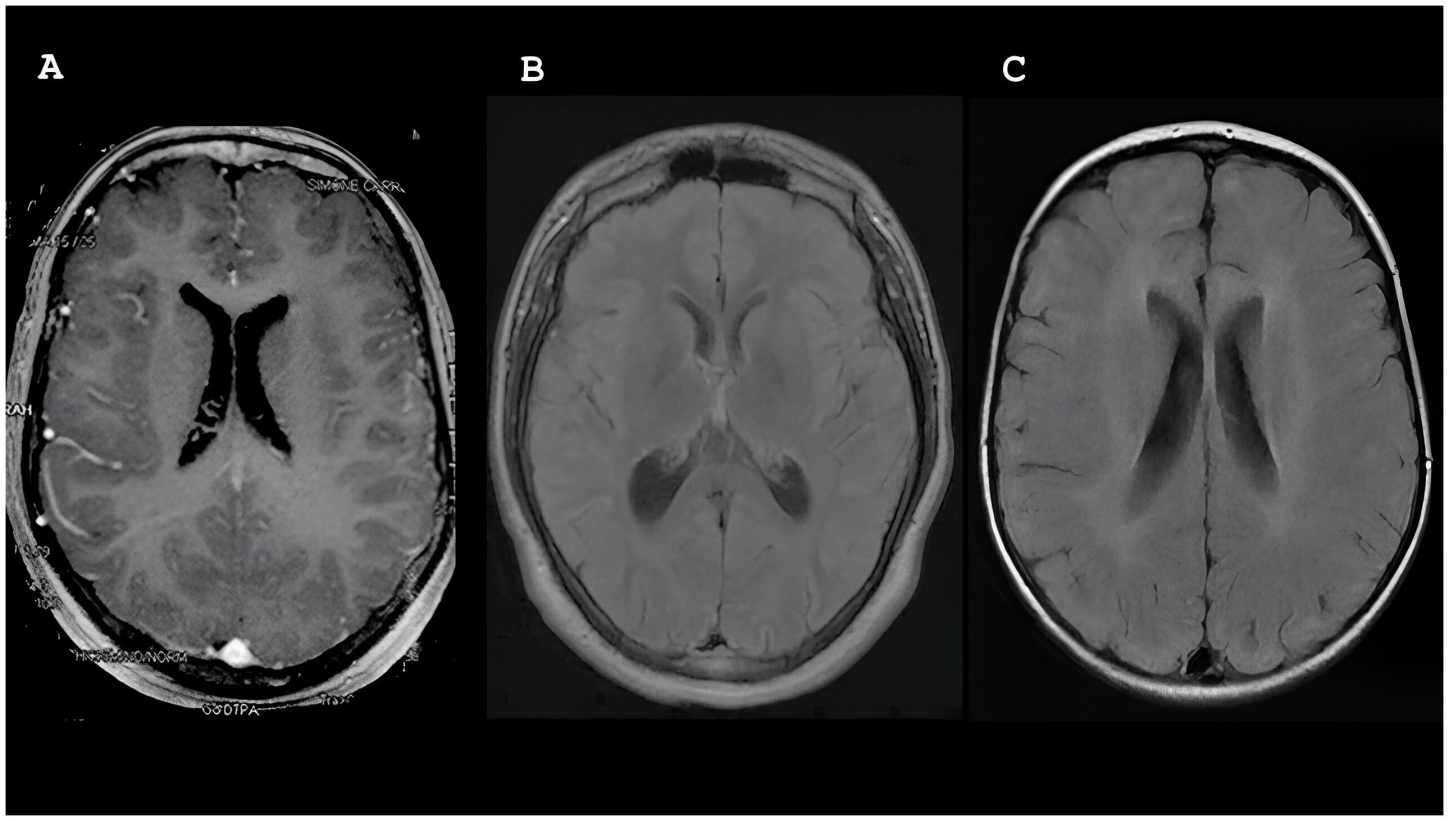
Magnetic resonance imaging (MRI) showing white matter changes in participants P4, P5 and P7.

Five patients (P2, P3, P4, P5, and P7) underwent brain magnetic resonance imaging (MRI). Among them, two (P2 and P3) displayed normal results, whereas the remaining three (P4, P5, and P7) showed abnormalities. Notably, two patients (P4 and P5) exhibited diffuse volumetric reduction in the brain, particularly in the cerebellar region. As shown in Figure 2, white matter changes were observed in three patients (P4, P5, and P7), with one patient showing peritrigonal hyperintensities (P4), another presenting scattered oval foci (P5), and the third (P7) displaying symmetrical bilateral involvement of both cerebral hemispheres with frontoparietal subcortical predominance.

Five mutations were identified in the *NPC1* gene: two missense (P1007A and A1035V), two frameshift (L1230fs and Q991fs), and one splice-site (IVS24 + 2 T > G) mutation(s). All newly described mutations have been predicted to be disease-causing according to the guidelines of the American College of Medical Genetics and Genomics (8).

Table 2 summarizes the behavioral and emotional problems evaluated in all patients. Patient (P5) presented attention problems and ADHD symptoms, P7 exhibited depressive problems, and P6 showed several psychopathological symptoms, including withdrawal, attention problems, thought disturbances, aggressiveness, rule-breaking behavior, and intrusiveness. Regarding the DSM-oriented scale, P6 showed symptoms of depression, avoidant personality, ADHD, and antisocial personality.

**Table 2:** Behavioral and emotional problems evaluation of patients with NPC.

		P1	P2	P3	P4	P5	P6			P7
ASR Syndrome Scale Scores (score T)	Anxious/depressed	52	51	51	50	50	61	CBCL Syndrome Scale Scores (score T)	Emotionally reactive	50
	Withdrawal	51	50	50	50	61	70		Anxious/depressed	56
	Somatic complaints	51	51	50	52	52	79		Somatic complaints	53
	Thought problems	55	50	50	56	50	90		Withdrawal	56
	Attention problems	59	52	50	50	73	73		Sleep problems	56
	Aggressive behavior	59	50	50	50	53	100		Attention problems	51
	Rule-breaking behavior	51	50	50	50	61	76		Aggressive behavior	50
	Intrusive	63	50	53	62	57	80			
ASR DSM-Oriented Scales (score T)	Depressive problems	50	57	50	50	59	65	CBCL DSM-Oriented Scales (score T)	Depressive problems	70
	Anxiety problems	51	50	59	50	50	60		Anxiety problems	54
	Somatic problems	51	50	50	56	50	75		Autism spectrum problems	58
	Avoidant personality	50	50	51	50	50	65		AD/H problems	50
	AD/H problems	54	50	50	50	70	85		Oppositional defiant problems	50
	Antisocial personality	56	50	50	50	53	86			
ASR	Internalizing problems	48	44	44	40	51	73	CBCL	Internalizing problems	53
	Externalizing problems	59	29	39	45	57	93		Externalizing problems	37
	Total problems	54	40	40	41	58	86		Total problems	49

NPC, Niemann-Pick disease type C; AD/H, attention-deficit/hyperactivity; CBCL, Child Behavior Checklist; DSM, Diagnostic and Statistical Manual of Mental Disorders; ASR, Adult Self-Report.

## 4 Discussion

Herein, we present a series of eight patients who were followed up since the onset of their first symptoms at the largest pediatric hospital in Brazil. We analyzed the clinical, neuropsychological, and genetic profiles of these patients, as well as their familial characteristics.

Ataxia is a cardinal and frequent symptom of NPC (14). Previous studies have reported improvements in ataxic symptoms following the initiation of miglustat treatment (15). However, in our study, only one patient showed sustained stabilization of their condition. The other two initially stabilized and then exhibited sequential progressive worsening. Given the small sample size, attributing this medication stabilization solely to transience is challenging and may represent an individual characteristic of this sample.

Neuroimaging in patients with NPC reveals a variable pattern, with some individuals showing normal results, whereas others exhibit the most common changes, such as cortical and cerebellar atrophy (16). The accumulation of lipid substrates within neurons leads to structural changes, including the formation of meganeurites, axonal distension, spheroid formation in axons, and ectopic dendritogenesis. Certain brain regions, such as the cerebellum, brain stem, hippocampus, and basal ganglia, are notably more affected in patients with NPC, resulting in neuroaxonal dystrophy due to ganglioside accumulation (17). These neuronal changes become evident during neuroimaging examinations as the disease progresses, manifesting as alterations in white matter integrity, myelination, and axonal integrity. Macroscopically, atrophy occurs in the cerebellum, thalamus, hippocampus, caudate, and cortical nucleus, which aligns with the MRI findings of the individuals in this study (18). Consistent with previous studies, imaging changes are correlated with increased phenotypic severity, particularly ataxia and ocular motor function (16, 19). These abnormalities may also be influenced by patient genotype.

Cognitive and/or behavioral impairments were identified at different severities in all patients. Cognitive impairment is consistently observed in patients with NPC, and its extent is directly proportional to the patient’s age and severity of clinical symptoms (5, 7). The pathogenicity of the variant correlated with both greater clinical severity and cognitive impairment. Of note, even in milder variants, the cognitive impact increased with advancing age.

Although psychotic symptoms are reported in approximately 25% of patients with NPC, some studies revealed observed rates as high as 55% (20–22). Notably, these patients often show resistance to antipsychotic medications, which may not be effective in managing these symptoms (23). Moreover, behavioral symptoms commonly associated with frontal dysfunction, such as hyperactivity, social cognitive impairment, disinhibition, and impulsive behaviors, are most prevalent in patients with NPC (22). Unlike cognitive impairment, the frequency of behavioral disorders did not increase in older patients despite signs of worsening as the disease progressed. Conversely, behavioral problems, particularly those related to aggression, were predominantly reported in patients with the A1035V variant, indicating a potential relationship between the type of mutation and observed behavioral disorders. Behavioral deterioration tends to occur in patients who are susceptible to this variant.

NPC manifests a broad phenotypic spectrum, encompassing variations in the age of symptom onset, disease progression rate, severity, affected organs, impact on the central nervous system, and response to pharmacological treatments (24). Moreover, the phenotypic expression of NPC can be influenced by factors such as the level of residual function of the defective protein and specific genetic variants involved. Cardinal symptoms primarily manifest as motor-related, behavioral, and psychiatric abnormalities that have a significant impact on the lives of patients and their families. Neurobehavioral and psychiatric manifestations, such as phenotypic severity and neuroimaging abnormalities, correlate with the patient’s genotype.

P1007A was initially described in Canadian patients (25) and is one of the most common “variant” alleles (16, 26). The P1007A mutation presents cellular changes typical of the so-called “variant biochemical phenotype,” characterized by normal low-density-lipoprotein (LDL)-induced cholesterol ester formation rate and minimal accumulation of non-esterified cholesterol in vesicles (27). A single P1007A allele is adequate for maintaining some degree of cholesterol trafficking, resulting in a variant phenotype typically diagnosed at a later age with lower biomarker levels (3, 27). Despite the observed intrafamilial phenotypic variability in patients with homozygous P1007A variants, all patients had NPCCSS scores below 9, with the highest score recorded in a patient aged >35 years.

A1035V appears as a relatively frequent allele in Portugal and Brazil (3, 27, 28). A1035V is a conserved mutation that partially affects the levels of mature mutant proteins, leading to a severe phenotype (29). Typically, it manifests with a classical phenotype (3, 27).

Patients with compound heterozygosity for the A1035V and P1007A variants scored <10; however, both patients experienced significant psychiatric and behavioral problems that were not identified in the other patients in this study. Psychotic symptoms and impulsivity are common manifestations of NPC (30). Although these findings were primarily observed in patients with the A1035V variant, they might not necessarily indicate a direct correlation between psychiatric or behavioral symptoms and the A1035V variant. Instead, milder clinical severity in patients with homozygous P1007A mutations or greater clinical severity in patients with loss-of-function or intronic variants could explain these findings.

Truncating mutations include nonsense, frameshift, and splice-site mutations (31), with one patient displaying a variant in the splice donor site with compound heterozygosity. Other variants of the splice donor site in intron 24 have been described in patients with NPC (26, 32). All truncating mutations are severe (31). In patient (P5), who possessed both a mild and a severe variant, a moderate phenotype was observed.

Other truncating mutations, specifically L1230fs and Q991fs, were identified, with both being uncommon variants that resulted in loss of function. As presented in this study, patients with loss-of-function variants had more severe forms (neonatal and early childhood) (33), with an earlier age of onset and a higher NPCCSS score. Although the classical definition of a severe phenotype typically implies the presence of severe mutations in both alleles (31), the described patients exhibited only one severe variant. This finding suggests that the presence of a single severe variant may be adequate for the development of an early and severe form of the disease.

Due to the rarity of the disease, this study was constrained by the small sample size and analysis conducted at a single site. A comprehensive approach involving clinical, genetic, and psychological data will contribute to a better understanding of this population.

## 5 Conclusion

NPC manifests diverse clinical presentations and consistently shows associations with cognitive and behavioral impairments. The severity of these impairments is correlated with age and specific genetic variants. Despite the small sample size, a subgroup showed a higher prevalence of psychotic and behavioral symptoms, suggesting a potential link with specific genetic variants. These insights highlight the multifaceted nature of NPC, in which cognitive and behavioral challenges are influenced by genetics, age, and cultural factors.

## Data Availability

The original contributions presented in the study are included in the article, further inquiries can be directed to the corresponding author.
